# BOLD: Bio-Inspired Optimized Leader Election for Multiple Drones

**DOI:** 10.3390/s20113134

**Published:** 2020-06-01

**Authors:** Rajesh Ganesan, X. Mercilin Raajini, Anand Nayyar, Padmanaban Sanjeevikumar, Eklas Hossain, Ahmet H. Ertas

**Affiliations:** 1 Department of Information Technology, MIT campus, Anna University, Chennai 600 044, India; gr@annauniv.edu; 2 Department of Electronics and Communication Engineering, Prince Shri Venkateshwara Padmavathy Engineering College, Chennai 600 127, India; raajii.mercy@gmail.com; 3 Graduate School, Duy Tan University, Da Nang 550000, Vietnam; anandnayyar@duytan.edu.vn; 4 Faculty of Information Technology, Duy Tan University, Da Nang 550000, Vietnam; 5 Department of Energy Technology, Aalborg University, 6700 Esbjerg, Denmark; san@et.aau.dk; 6 Oregon Renewable Energy Center (OREC), Department of Electrical Engineering and Renewable Energy, Oregon Tech, Klamath Falls, OR 97601, USA; 7 Department of Mechanical Engineering, Faculty of Engineering & Natural Sciences, Bursa Technical University, Bursa 16330, Turkey; ahmeth.ertas@btu.edu.tr

**Keywords:** unmanned aerial vehicle, multiple UAV, clustering, leader election, drones, particle swarm optimization, spider monkey optimization, network lifetime

## Abstract

Over the past few years, unmanned aerial vehicles (UAV) or drones have been used for many applications. In certain applications like surveillance and emergency rescue operations, multiple drones work as a network to achieve the target in which any one of the drones will act as the master or coordinator to communicate, monitor, and control other drones. Hence, drones are energy-constrained; there is a need for effective coordination among them in terms of decision making and communication between drones and base stations during these critical situations. This paper focuses on providing an efficient approach for the election of the cluster head dynamically, which heads the other drones in the network. The main objective of the paper is to provide an effective solution to elect the cluster head among multi drones at different periods based on the various physical constraints of drones. The elected cluster head acts as the decision-maker and assigns tasks to other drones. In a case where the cluster head fails, then the next eligible drone is re-elected as the leader. Hence, an optimally distributed solution proposed is called Bio-Inspired Optimized Leader Election for Multiple Drones (BOLD), which is based on two AI-based optimization techniques. The simulation results of BOLD compared with the existing Particle Swarm Optimization-Cluster head election (PSO-C) in terms of network lifetime and energy consumption, and from the results, it has been proven that the lifetime of drones with the BOLD algorithm is 15% higher than the drones with PSO-C algorithm.

## 1. Introduction

**Figure 1:**
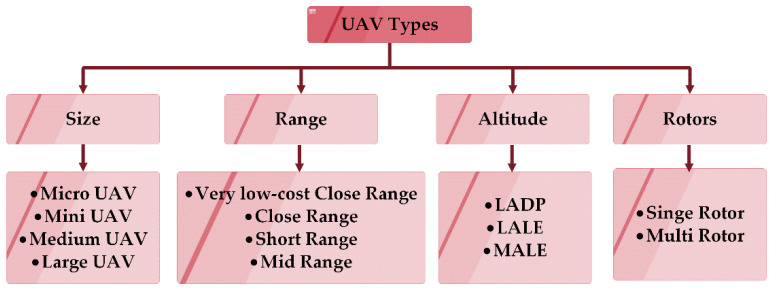
Classifications of Unmanned Aerial Vehicle types drones based on size, range, number of rotors, and altitude.

In the past, drones or unmanned aerial vehicles (UAVs) have been used only as expensive military aircraft or small toys for kids. After the expanded permission of the federal aviation administration for using drones for commercial and non-hobbyist purposes, drones are being applied in day to day activities. Drones replace the traditional method of business operations with less human and limited infrastructure, which helps to reduce the time and cost of commercial and business activities. As a consequence, drones are widely applicable in various domains such as in agriculture for crop and herd monitoring, environmental and natural disaster monitoring, aerial photography, border surveillance, emergency assistance, search and rescue missions, relay communications and weather monitoring [1,2]. The need for drones has increased linearly over the past decade. According to the Drone Market Report 2019, by the Drone Industry Insights, the sales of the commercial drones are expected to increase and India will become the third-largest commercial drone market in the year 2024 [3]. Drone-based communication systems provide two kinds of communication—air to ground (communication with a base station) and air to air (communication with other drones) [4]. The recent development in drones and its related technologies opens the market for various commercial applications. Based on the application or problem to be solved, the drones of various types can be applied. In general, drones can be classified based on their size, range, and endurance, number of rotors, and altitude, as shown in Figure 1 [5].

UAV network topology is highly complex due to the dynamic, three-dimensional (3D) environment with changing UAV velocities. Another difficulty is the limitation of the range between UAVs and the ground station. When the number of UAVs increases, it is essential to use clustering schemes as they ensure the necessary level of network performance, such as end-to-end delay, throughput, and energy efficiency. The UAVs are divided into multiple groups or clusters in the clustering technique, which shares the same geographic location. For such a clustered setup, only the chosen cluster head (CH) is responsible for inter-cluster and intra-cluster communication [6,7].

**Figure 2:**
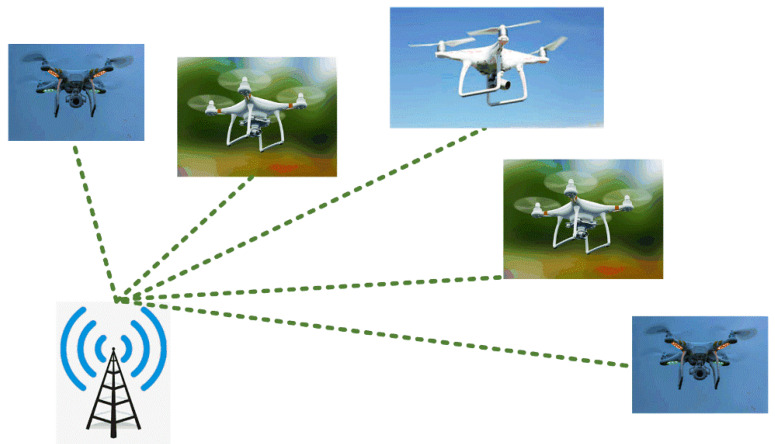
Generic drone communication architecture illustrating the communication between each drone and base station.

Usage of single drones for the applications mentioned above limits the efficiency of missions and could not be achieved in some cases. In such cases, multiple drones are preferred over a single drone for successful mission completion. Hence, the need for efficient communication among multiple drones arises. For the better achievement of this, a leader is elected to co-ordinate the multiple drones. However, implementing multiple drones in real-time is not as easy when compared to a single drone because, in the case of multiple drones, many critical factors come into consideration like communication between the drones and range of communication. To be more precise, controlling multiple drones in performing a single operation is a hideous process because if there is no proper communication between multiple drones, there is the chance that one drone may collide with the other [8]. Further, to improve efficiency in battery capacity and to perform separate tasks, multiple drones are divided into clusters. Even in these clusters, leaders are elected for better communication among drones. Figure 2 depicts the schematic representation of each drone communicating with the base station. Leader election and cluster formation are done based on the bio-inspired optimizations such as PSO and SMO, respectively.

Researchers have proposed many bio-inspired optimization algorithms to solve complex computational problems. They are stimulated or inspired by the biological behavior of animals or birds. The optimal solution is found by exploring and exploiting the search spaces using different methods [1]. The widely used algorithms include Particle Swarm Optimization, Grey Wolf Optimization, Ant Colony Optimization, Artificial Bee Optimization, and so on [9,10,11,12]. Particle Swarm Optimization (PSO) proposed by Eberhart and Kennedy, which is derived from the study on flocking of birds or fish schooling ich behavior of animals and considers an example that a group of birds is searching for a single piece of food in a random area [13]. Every bird does not know the location of the food particle, but it can be found out in repeated iterations. Thus, the practical strategy is to find the bird which is closest to the food. In the PSO, random particles (solutions) are first initialized, and an optimal solution is found in successive iterations [13]. The Spider Monkey Optimization (SMO) technique uses the social behavior of spider monkeys for solving optimization problems. It is based on a fission-fusion methodology. Generally, they live in large groups of individuals. If there is a need, they separate (fission) from the group and reunite (fusion) later when needed. This method is widely used for clustering techniques [14].

The effective utilization of battery energy is one of the most crucial factors for the operation of the drones. The battery power in the drones is used for wireless communications between the drones and to the base station, data processing, drone hovering, and various other purposes depending on the application of the drone. This requirement gives rise to the design and implementation of an energy-efficient algorithm for real-time processing of drone data. In the case of multiple drones, numerous communication and coordination challenges need to be solved. The high velocity of the drones ranging from 35 km/h to 70 km/h is another challenge because collisions can be avoided only when the obstacle avoidance algorithm is executed immediately [15]. For this purpose, drones need to make formations according to their flying environment. The research gaps that need to be solved are:

• When multiple drones are deployed in the environment, communication among the drones and communication with the base station takes place.
• If each drone communicates separately with the base station, more energy will be consumed, and thus the lifetime of the network may decrease.
• When the communication between the drones fails, then collision may occur as the drones are not aware of their neighbor’s position.

Our contributions to overcome the problems above are:

• A cluster head CH is elected dynamically based on its current position (nearer to the base station and all other drones),residual energy (b_energy_), and velocity using a method based on Particle Swarm Optimization (PSO).This elected leader CH alone communicates with the base station and other drones, thereby decreasing the communication energy, which in turn extends the network lifetime.
• Clusters are formed using Spider Monkey Optimization (SMO) based on the proximity of the drones to each other, its connectivity to other nodes (using RSSI), and the residual energy of the drones. The clusters formed will have equal average residual energy for increasing the network lifetime furthermore.
• Simulation of the proposed algorithm, to compare and study its efficiency with the existing algorithm.

Our assumptions are as follows:

• The network model is initially considered to have homogenous drones with the same amount of residual energy, and their moving direction is random.
• The drones used are attached with a global positioning system(GPS) from which its position can be calculated at any instance and thereby calculating the distance traveled by it at the given period.
• Communication cost among the drones is considered to be high rather than the computation cost between the drones and the base station.
• The algorithm might not run continuously, but only when either the battery of the leader is less than 40% or when there is an extreme change in the network topology or connectivity.
• The drone *d* is initially elected as the leader at the ground level, and after some time, the leader election may take place among the clusters in the flying environment.
• The target for all the drones is considered to be the same. Sometimes, when multiple targets are considered, they are assumed to be nearby.

The rest of the paper is arranged as follows: Section 2 draws a literature survey focusing on the present research works. In Section 3, the proposed work is illustrated with necessary system architecture and mathematical modeling. The proposed BOLD algorithm is discussed in detail in this section, as well. The implementation of the proposed algorithm based on which the performance evaluation is drawn can be found in Section 4. Finally, the study is concluded in Section 5 with a conclusion.

## 2. Literature Survey

In earlier literature, motifs, as a basic unit for mission planning, were proposed by Liu, J. et al. [15], which, was based on dynamic reconfiguration since UAV swarm communication with limited resources was difficult. Using the solution of motif-based swarm configuration, they used a multidimensional dynamic list scheduling algorithm to create a mission planning scheme. Dynamic topology has been used by Flying ad hoc networks (FANETs). However, UAV’s limited battery power and mobility cause unstable routing within the FANET. Khan minimized this issue [16] with the aid of an optimal clustering method. The authors have suggested a bio-inspired clustering scheme for FANETs (BICSF), which combines both the glow-worm swarm optimization (GSO) and krill herd (KH) method. For optimizing the consumption of energy and the election of fixed group leader, a framework for unmanned aerial vehicle wireless sensor networks (UAV-WSN) using weighted k-means cluster and simulated annealing (WKMC-SA) was developed by Hui-Ru Cao et al. [1]. The WKMC was used to group land WSN and assigned leaders of the group as fixed nodes to reduce the consumption of energy and retransmission rate. The SA algorithm was used to optimize the flight path planning. However, the group leaders were stationary re-election of a new leader in case of failures were not addressed by the author.

An algorithm that detects the formation of multiple UAVs was developed by Wang, Y. et al. [17]. The algorithm used here is the Weighted Component Stitching (WCS) algorithm. Here the formation of a network was calculated using the inter UAV distance with the help of the Ultra-Wide Band (UWB) module. However, this became difficult when the network becomes sparse and noisy. The problem, as mentioned above, was solved by reliable grouping components of a graph into groups and thereby calculating the formation of more extensive and sparse networks with accuracy. Further, for tracking the formation over a while, the Kalman filter was integrated into the WCS algorithm. These algorithms were compared with existing ARAP (as rigid as possible), ASAP (as soon as possible), and SDP (semi definite programming) algorithms. The series of results showed that WCS converged much quicker than ARAP; however, complexities remained the same. Tracking simulation also proved that WCS and WCKF had better accuracy than that of the existing approaches. A method that is based on a distributed consensus-based algorithm which combines the sensing capabilities was proposed by Tao Feng et al. [18]. Cluster heads communicate within the cluster or with cluster heads of other clusters using frame relay. Homogeneous UAV clusters have the same communication distances and capacities. The above-mentioned problem was simplified by assuming the capacity will not change with respect to its location and remains constant for all directions. This method gives an extensive knowledge about the state transitions of UAVs and also improves the coordinated control of UAV cluster. The feasibility of the consensus-based algorithm was performed by repeated simulations and proves that the algorithm is scalable and adaptable.

Spider Monkey Optimization (SMO) is an algorithm inspired by the social Fission-Fusion (FFS) structure of spider monkeys during their foraging activity. Due to its high efficiency, the algorithm was used to solve complex problems. [14]. Spider monkey optimization has been applied in various fields like big data [19], image processing [20], cluster routing protocols for WSNs. In Reference [21], research was carried out to understand the mechanism of SMO in WSN route optimization by implementing a mathematical model of its behavioral patterns based on SMO-C. It transmits the data from the clusters to the base station via the optimal path. Furthermore, the SMO-C is considered to be better than the conventional routing protocols in terms of less energy usage and better network service efficiency. A Hybrid Particle Swarm Optimization and Genetic Algorithm (HPSOGA), which solved the multi-UAV formation reconfiguration problem, was proposed by Duan et al. [22]. This new approach, when combined with the Particle Swarm Optimization (PSO) and Genetic Algorithm (GA), finds the time-optimal solutions. The HPSOGA was compared with the simple PSO algorithm, and the results showed that the former is better than the later in solving both multi-UAV formation reconfiguration problems and finding time-optimal solutions under complicated environments.

PSO-C, an energy-aware cluster head election using the PSO algorithm, was proposed by Latiff et al. [23]. This algorithm optimized the network energy consumption using intra-cluster distance and ratio of initial energy to the total energy of the network. However, in some cases, non-cluster heads were assigned as cluster heads in the clusters formed, which decreased the lifetime of the network due to insufficient energy consumption, and sink distance was not used in fitness computation. The PSO-C algorithm was compared with LEACH (Low Energy Adaptive ClusteringHierarchy) and LEACH-C (Low Energy Adaptive Clustering Hierarchy-cluster based protocol). J. Sanchez-Garcia et al. [24] proposed a novel dynamic Particle Swarm Optimization for UAV networks (dPSO-U). This dPSO-U was for a path optimization in the rescue operations of the given disaster situation. It was compared to the optimal trajectory planning algorithm, and the results were found to be more efficient than that.

Particle Swarm Optimization is a swarm intelligence algorithm based on birds flocking behavior. Let N_P_ be the number of particles in PSO. A particle P_i_ has position ***X_i_*** and velocity ***V_i_***. The fitness function is used to evaluate each particle for checking the quality of the solution. Initially, each particle is assigned with a random position and velocity values. Each particle computes its own best called ***Pbest_i_*** and global best called ***Gbest*** for every iteration. To reach the global best solution, it uses its personal and global best to update the velocity ***Vi*** and position ***Xi*** using the following Equations (1) and (2).

(1) $$V_{i}\left(t+1\right)=w\times V_{i}+c_{1}\times \chi_{1}\times \left(X_{Pbest_{i}}-X_{i}\right)+c_{2}\times \chi_{2}\times \left(X_{Gbest}-X_{i}\right)$$

(2) $$X_{i}\left(t+1\right)=X_{i}+V_{i}\left(t+1\right)$$

where ***w*** is the inertia weight, ***c***_1_, ***c***_2_ are acceleration coefficients and $\chi_{1},\;\chi_{2}$ are randomly generated values.

(3) $$Pbest_{i}=\{\begin{array}{c} P_{i,\;}if\;(Fitness\left(P_{i}\right)<Fitness\left(Pbest_{i}\right)\\ Pbest_{i},\ldots otherwise \end{array}$$

(4) $$Gbest=\{\begin{array}{c} P_{i,\;}if\;(Fitness\left(P_{i}\right)<Fitness\left(Gbest\right)\\ Gbest,\ldots otherwise \end{array}$$

After getting a new updated position, the particle evaluates the fitness function and updates ***Pbest_i_*** as well as ***Gbest*** from Equations (3) and (4).

Stochastic optimization was proposed by modeling the social behavior of spider monkeys foraging. Spider monkeys have been categorized as fission-fusion social structure based animals. The animals which follow fission-fusion social systems, initially work in a large group and based on need after some time, they divide themselves into smaller groups led by an adult female for foraging.

Initially, the SM group starts food foraging and approximates their own distance from the food. Next, based on the distance from the foods, group members update their positions and again evaluate the distance from the food sources. Furthermore, in the third step, the local leader updates its best position within the group and if the position is not updated for a specified number of times then all members of that group start searching for the food in different directions. Finally, in the fourth step, global leader, updates its best-ever position and in case of stagnation, it splits the group into smaller size subgroups. All the four steps mentioned aforesaid, are continuously executed until the desired output is achieved. The population initialization, local leader election and global leader election are given by Equations (5)–(7) respectively.

(5) $$spm_{xy}=spm_{\min y}+v\left(0,1\right)\times \left(spm_{maxy}-spm_{miny}\right)$$

where $spm_{xy}$, is $spm_{x}$ in the yth direction, range of ***v*** between 0 to 1; $spm_{maxy}\;and\;spm_{miny}$ are minimum and maximum bounds.

(6) $$spm_{newxy}=spm_{xy}+v\left(0,1\right)\times \left(Lbest_{kj}-spm_{xy}\right)+v\left(-1,1\right)\times \left(spm_{ry}-spm_{xy}\right)$$

(7) $$spm_{newxy}=spm_{xy}+v\left(0,1\right)\times \left(Gbest_{y}-spm_{xy}\right)+v\left(-1,1\right)\times \left(spm_{ry}-spm_{xy}\right)$$

$spm_{newxy}$ is the new location of spm; Lbest and Gbest are the local and global leaders of the population.

From the literature, it has been observed that in recent years, the multiple drones are widely used in many applications, but still, some significant aspects like clustering, cluster head election are not considered when multiple drones are deployed for single target events in the environment [25,26,27,28]. The re-election of cluster heads in case of failures was also not considered. Network lifetime is a significant parameter in any ad-hoc and mobile networks when a substantial amount of nodes in the network dies; there is a high probability of network disconnection, which reduces network lifetime. Another challenge in the highly dynamic networks like UAV is topology control; when a node moves far away from the network coverage, it will affect the topology and the process of achieving the target. The main idea of our proposed algorithm is the election of cluster head and formation of clusters, a hierarchical topology network, which can minimize the energy consumption and maximize the network lifetime. In the hierarchical clustering approach, the nodes are grouped into clusters or again as sub-clusters, a cluster head(CH) is elected. The CH is responsible for the coordination of the cluster members(CM), intra-cluster, and inter-cluster communications. The clustering approach minimizes the communication overhead eliminates the communication between CMs and the base station(BS), which will extend the network lifetime, increase network scalability, and also reduce routing overhead. Later sections will provide a solution to these problems.

## 3. Proposed Work

### 3.1. System Architecture

**Figure 3:**
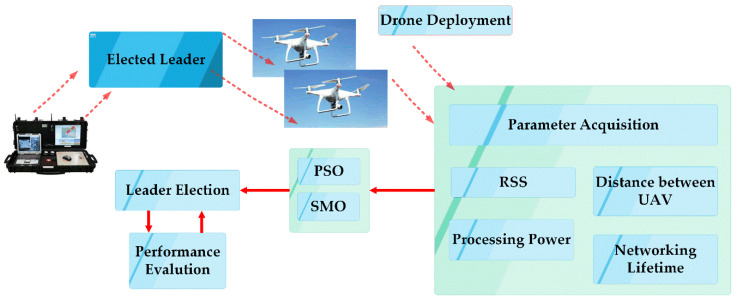
The system architecture of proposed BOLD for multiple drones.

The architecture of the proposed work, BOLD, is shown in Figure 3, which represents that when multiple drones are deployed, network parameters like energy, the distance between drones and processing power are taken into consideration and using Algorithm 1 (BOLD algorithm).Based on the mentioned parameters, the fitness function is computed for each node, and the best or optimized leader is elected as CH, and this elected leader communicates with the base station and all other drones in our model. Iteratively, the network undergoes re-evaluation, which triggers the BOLD algorithm to elect new CH.

### 3.2. Mathematical Models of BOLD

**Figure 4:**
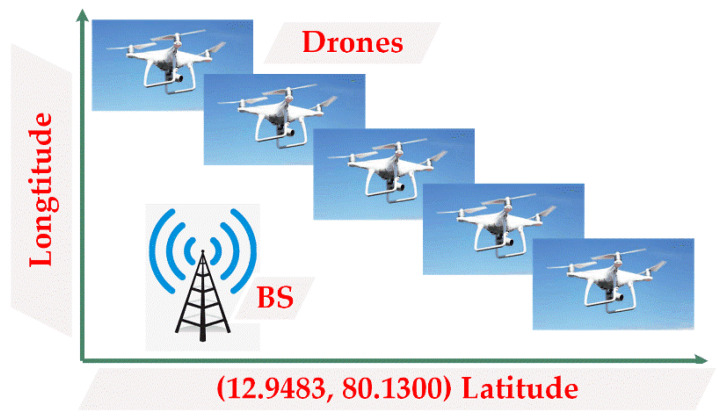
Initialization of drones by assuming the location of the Base station at a predetermined location.

Let the model consist of *n* drones. The battery and positions of each drone are initialized based on the real-time values. The initial position of the drones are assumed to be in between the co-ordinates of 10.000° N–25.500° N (latitude) and 80.000° E–95.500° E (longitude) and their flying direction is considered to be random. The base station is assumed to be at the location 12.9483° N and 80.1399° E, as shown in Figure 4.

The fitness value (*B_opt_*) for each drone is calculated using Equation (8).

(8) $$Fitness\;value\left(B_{opt}\right)=\left(k\times d_{avg}\right)+\left(0.5\times b_{energy}\right)$$

where,

$d_{avg}$ is the average distance between the nodes

$b_{energy}$ is the residual energy

*K* is a variable ranging in value from 0.2 to 0.4

The value of k is computed experimentally to range from 0.2 to 0.4 because, in this range, the leader elected is said to be more optimized and capable of running for more iterations.

The average distance *d_avg_* between the nodes is computed to find the drone which is nearer to the base station and to all other drones using Equation (9).

(9) $$d_{avg}=\frac{d_{1}+d_{2}+\ldots +d_{n}+d_{BS}}{n+1}$$

where,

*n* is the number of nodes

*d_BS_* is the distance between drone and base station.

**Figure 5:**
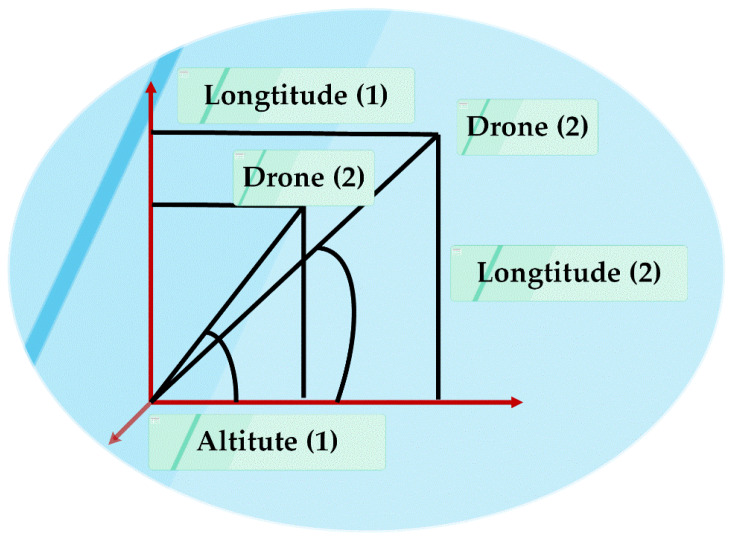
3D representation of the haversine formula for determining the distance between two drones.

After computing the fitness value (*B_opt_*) of each drone at time t, the position and the battery values of the drone are updated using the below-mentioned formulas and after updating, the fitness value (*B_opt_*) at time t + 1 is calculated again for all the drones. The Haversine formula calculates the distance(d) between two drones in 3-dimensional space as Equation(10) [29].The diagrammatic representation is shown in Figure 5.

(10) z=R×c

where,

*R* is the radius of the earth (6371 km)

*c* is computed by the formula

(11) $$c=2\times a\times \tan\;2\left(\sqrt{a},\sqrt{(1-a}\right))$$

(12) $$a=\sin^{2}\left(\frac{\Delta \varphi }{2}\right)+\cos\left(\varphi_{1}\right)\cos\left(\varphi_{2}\right).\sin^{2}\left(\frac{\Delta \lambda }{2}\right)$$

where,

(13) $$\Delta \varphi =\varphi_{2}-\varphi_{1}$$

(14) $$\Delta \lambda =\lambda_{2}-\lambda_{1}$$

where,

φ is the latitude value for the drone

λ the longitude value for the drone

After calculating the value of z, we used Equation (15) to determine the distance between two drones based on the altitude. This distance (d) is used in Equation (8) for calculating the distance between the nodes.

(15) $$d=\sqrt{z^{2}-\Delta {\mathrm{alt}}^{2}}$$

(16) $$\Delta \mathrm{alt}={\mathrm{alt}}_{2}-{\mathrm{alt}}_{1}$$

Since the energy of drones decreases with time, we used the energy model Equation (17) to calculate the updated battery value for every iteration.

(17) $$b_{energy}=b_{energy}-\left(l\times E_{elec}+l\times E_{amp}\times d^{2}\right)$$

where,

*l* is the number of bits transmitted per second

*d* is the distance between the drones

*E_elec* is the energy dissipated

*E_amp* is the amplifier energy to update the position of each drone, we add the distance covered by them with their current position. To calculate the distance covered we used Equation(18).

(18) $$\mathrm{Distance}=\frac{\mathrm{Speed}}{\mathrm{Time}}$$

The average range of velocity of drones deployed was from 58 to 61 mph.

The position shift of the drones was updated by updating the latitude and longitude positions using (19) and (20),

(19) λ=λ+distance

(20) φ=φ+distance.

The new latitude (φ) and longitude (λ) were again used in Equations (13) and (14) to calculate the distance of the drones.

### 3.3. BOLD Algorithm

**Figure 6:**
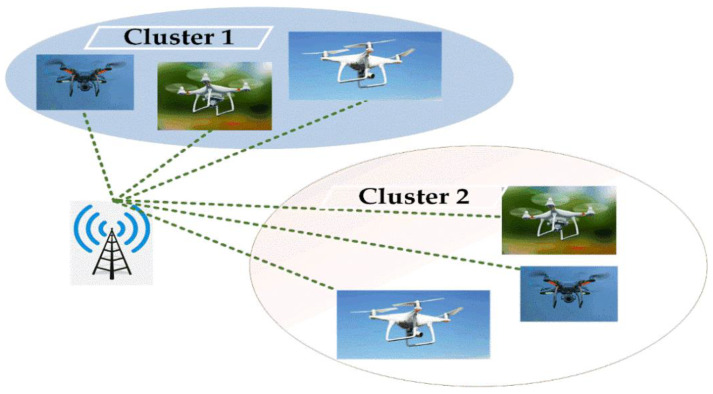
Clustering architecture in BOLD.

**Figure 7:**
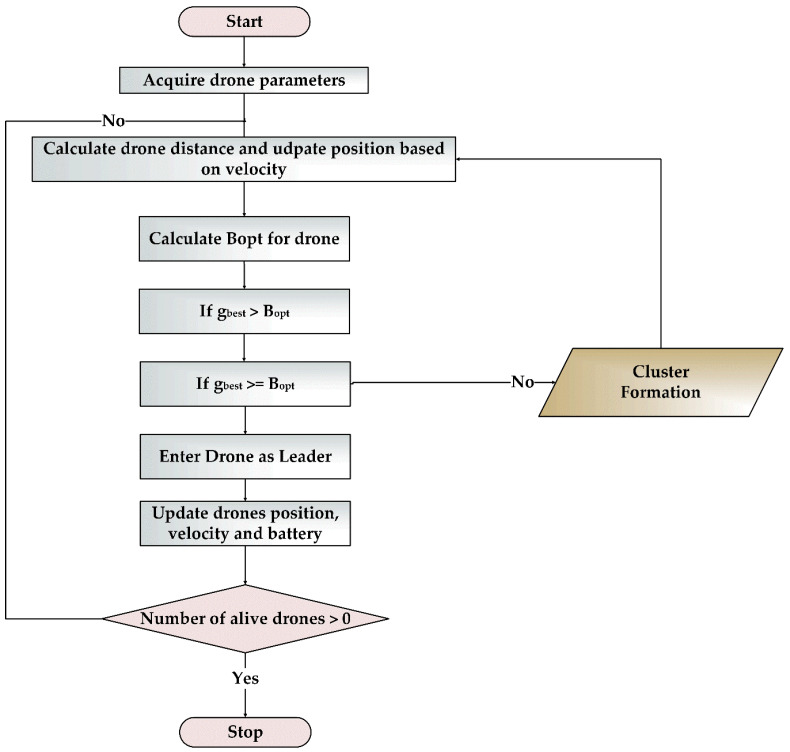
Flowchart of the proposed BOLD algorithm.

The BOLD algorithm can be divided into two phases, namely leader election, and cluster formation. In the leader election phase, the distance and residual energy of the drone were taken as the parameters for selecting the leader. For cluster formation, as shown in Figure 6, the drones were grouped into clusters based on their proximity and their residual energy. The overall algorithm is proposed in Algorithm 1, and the flowchart is shown in Figure 7. The description for leader election and cluster formation is explained below.
**Algorithm 1.** BOLD ALGORITHM**Let drone d initially be the leader with fitness value***gbest*.**for***each drone***do**Initialize each drone with its position and $b_{energy}$;**end**Divide *drone_i….k_* into smaller groups;**for** each group **do****for***drone_k_***do**Calculate the distance of *drone_k_* from other drones;Update *drone_k_* position;Calculate $B_{opt}$ value;**if**$B_{opt}$ ≥ *gbest*
**then***gbest* = $B_{opt}$;**end**Elect *drone_k_* as leader;**end****end****for***each drone***do**Calculate drone *velocity*;Update drone *position*;Update drone $b_{energy}$ using Equation (17);**end**

The main objective of the proposed algorithm is to elect an energy-efficient leader CH to extend the lifetime of the network. For this, residual energy of the drone and distance parameters such as the distance of the drone from the base station and distance between the two drones was considered. Let *B_opt_* be the fitness value for the particular drone calculated using Equation (8). This calculation is carried out in loop for each drone, and if the present *B_opt_* value is higher than the previous *B_opt_* value, then the current *B_opt_* value is taken as the *gbest*. At the end of the iteration, the *gbest* is optimized fitness value of the drone to be elected as the leader. After some time, the residual energy decreased based on Equation (17). For the next iteration, the updated position and residual energy value are considered for leader election. The algorithm for leader election is given in Algorithm 2.
**Algorithm 2.** LEADER ELECTION ALGORITHM**for***every iteration***do****for***drone_i_***do**  Calculate distance of *drone_i_* from other drones;  Update *drone_i_* position from GPS;Update *node velocity;*  Calculate ***B****_opt_* value;  **if**
*B_opt_* ≥ *gbest*
**then**   *gbest = B_opt_*;  **end**  Elect *drone_i_* as leader;**end****for***each drone***do**  Calculate drone *velocity*;  Update drone *position*;  Update drone $b_{energy}$;**end****end**

The cluster formation is based on the node’s proximity and the residual energy value of the drones. When drones closer to each other are assigned to the same cluster, then the transmission range will be less and thereby decrease energy consumption. Moreover, cluster formation takes place in such a way that the average residual energy of the clusters is equal. The residual energy is sorted in ascending order and is divided into clusters where each cluster as the same average residual energy value. The algorithm for cluster formation is given in Algorithm 3. Then, the leader election is performed again on these clusters separately using the leader election algorithm. Hence, the new leader is elected for each iteration based on the formulas given above. This is an elected leader from each cluster will communicate with the base station.
**Algorithm 3.** CLUSTER FORMATION**for** i: 1 to Nj = i;**while**(j > 1 &&$b_{energy}$ (j − 1) >$b_{energy}$ (j))swap($b_{energy}$ (j), $b_{energy}$ (j − 1))swap(lat(j), lat(j − 1))swap(long(j), long(j − 1))swap(alt(j), alt(j − 1))j = j − 1;**end****end****for all drones n :**q = n/4;h = n/2;hq = h+q;arr1 = arr(1:q);arr2 = arr(q + 1:h);arr3 = arr(h + 1,hq);arr4 = arr(hq + 1,n);optarr1[arr1 ][arr4 ];optarr2[arr2] [arr3 ];**end****for** each cluster **do****for***drone_k_***do**Calculate distance of *drone_k_* from other drones;Update *drone_k_* position ;Calculate $B_{opt}$ value;**if**$B_{opt}$ ≥ *gbest*
**then***gbest* = $B_{opt}$;**end****end**Elect *drone_k_* as leader;**end**

## 4. Implementation and Performance Evaluation

This section discusses the simulation and analysis of the proposed work’s results. The simulation was done using MATLAB and parameters like, (i) energy consumption vs. the number of drones, (ii) residual energy vs. the number of iterations, (iii) number of alive nodes vs. the number of iterations, (iv) network lifetime vs. the number of drones, (v) cluster building time vs. the number of nodes were plotted and analyzed by comparing the proposed BOLD and PSO-C.

### 4.1. Simulation Setup and Network Topology

**Figure 8:**
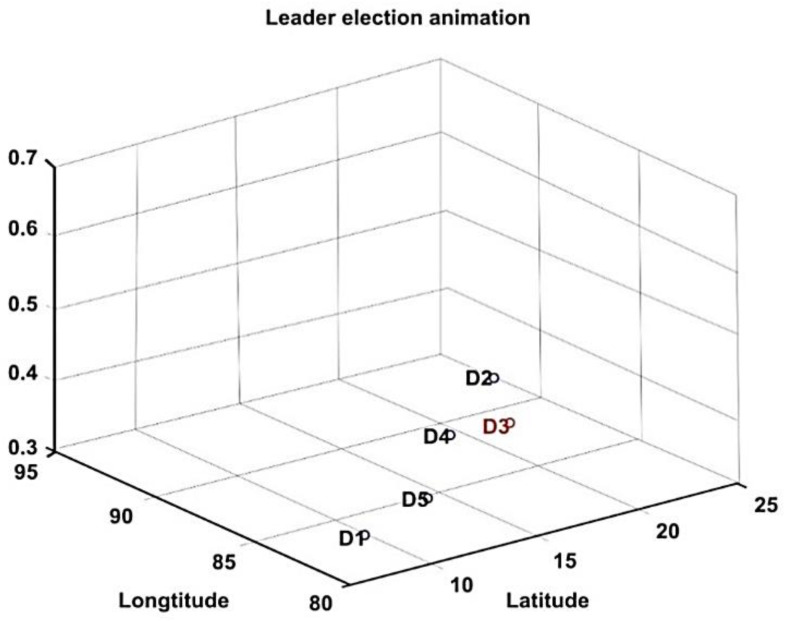
Network topology with 5 drones and the red color denotes the CH.

**Figure 9:**
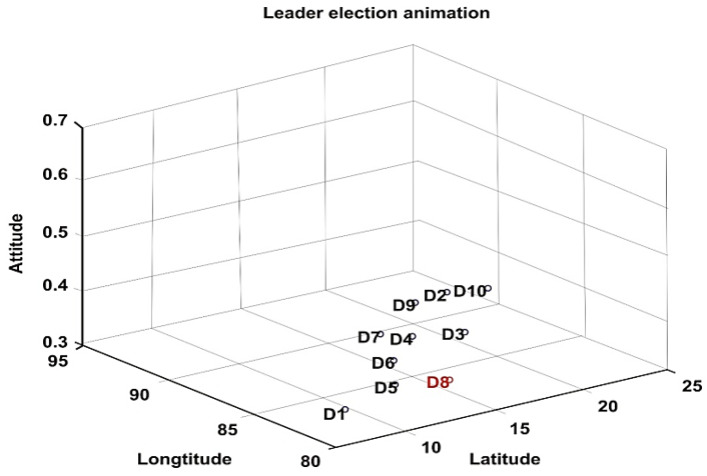
Network Topology with 10 drones with elected CH.

**Figure 10:**
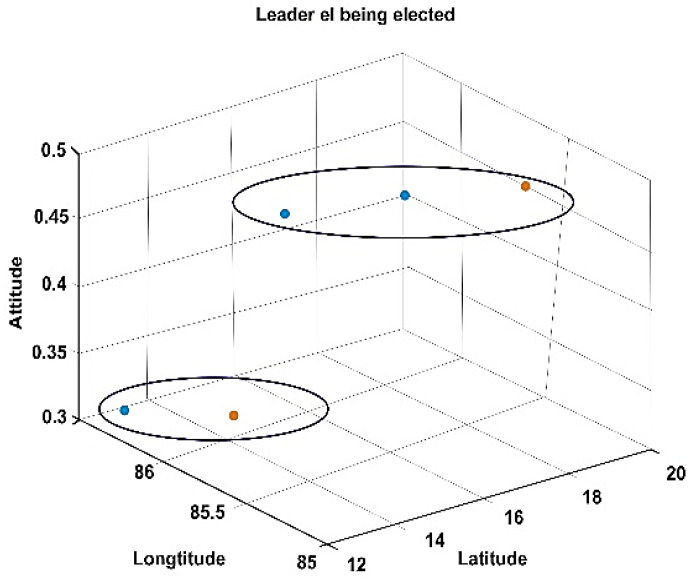
Representation of clustering of five drones as two clusters.

The proposed methods were simulated using the MATLAB 2018a version. MATLAB is a multi-paradigm numerical computing environment developed by MathWorks. Network simulation parameters and respective specifications are shown in Table 1. As a study, the deployment and movement of five drones and 10 drones concerning latitude, longitude, and altitude are shown in Figure 8 and Figure 9, respectively. The CH being elected at that particular instance is highlighted in red. The two cluster formation is represented in Figure 10 where cluster 1 has three drones while cluster 2 has two drones.

The data and computation results of the finding distance and optimal fitness value, as mentioned above, are tabulated in Table 2 and Table 3.

The initial values of the five drones are specified in Table 2. For iteration 1, the distance calculated using Equation (9) and *B_opt_* value using Equation (8) is specified below in Table 3.

Since the *B_opt_* value of drone 5 is higher than the rest of the drones, D1 is selected as the leader for the first iteration. Before the next iterations begin, the latitude, longitude, and battery values of the drones are updated using Equations (20), (19), (17) respectively.

**Figure 11:**
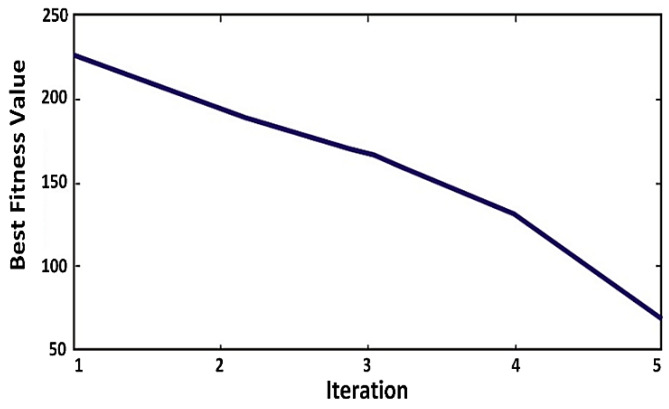
Reduction of best fitness values (*B_opt_*) with each progressing iteration caused by decreased battery values.

In Figure 11, the fitness value (*B_opt_*) of the drones is plotted, which decreases with an increase in the number of iterations. It is because the battery value decreases after each iteration, and the position of the drone also changes.

### 4.2. Result and Discussions

**Figure 12:**
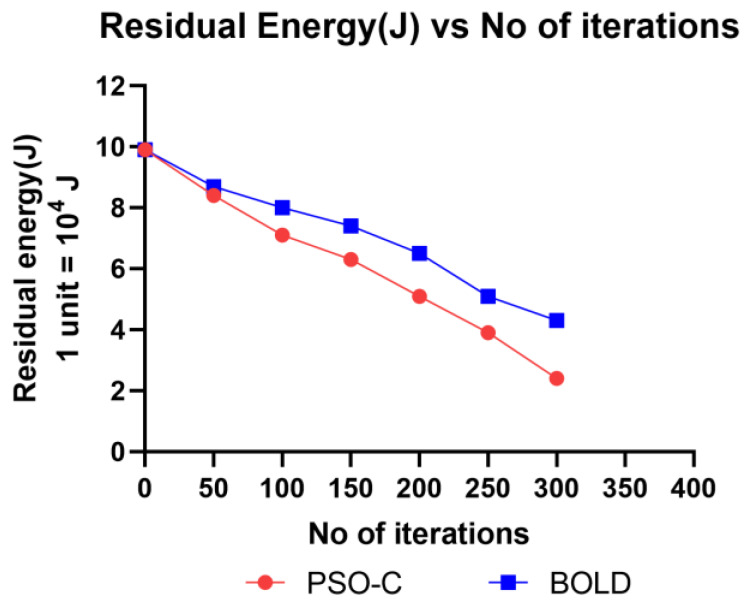
Residual energy vs each iteration.

**Figure 13:**
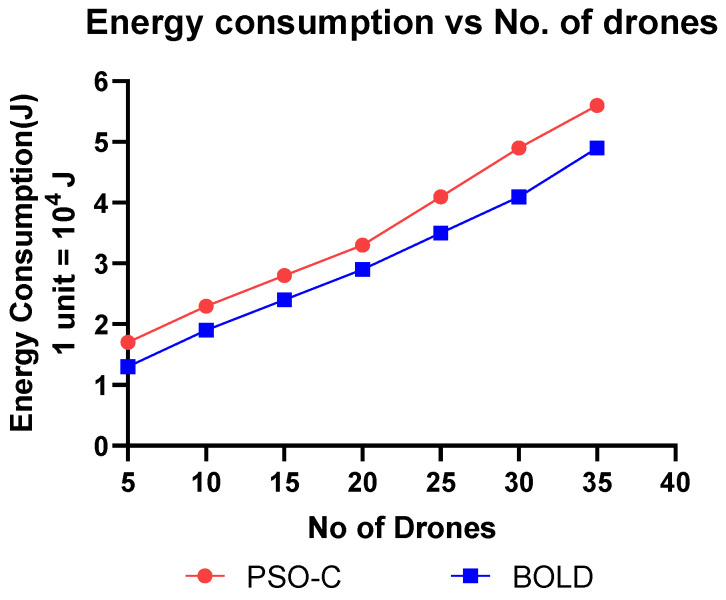
Energy consumption vs. the number of drones.

In mobile ad-hoc networks, most of the battery power of the nodes are consumed for communication than the computation and operation. Drones are high mobility nodes, which spent most of their battery energy not only for communication but also operation like flying and balancing themselves in the atmosphere. Thus, when the leader CH is elected, it takes charge of communication so that the energy is consumed less by other CM, which in turn increases the lifetime of the drones. The observed sample values residual energy after some iterations are given in Table 4 and plotted in Figure 12. From both, it is observed that the residual energy of the nodes decreases after some iterations of CH elections. Figure 13 and Table 5 represent the average energy consumption is increases concerning network scalability. The proposed BOLD performs better in average energy when compared to PSO-C. This is because only the elected CH drone communicates with the base station and so the energy consumption is reduced for all other drones. Since the CH is elected dynamically, the energy value of all drones in the network decreases gradually. The energy consumption of BOLD is 0.5% less than the PSO-C. In battery-operated drones, the maximum fly time will be from 30 to 45 min, the 0.5 % of each node will extend the network lifetime.

**Figure 14:**
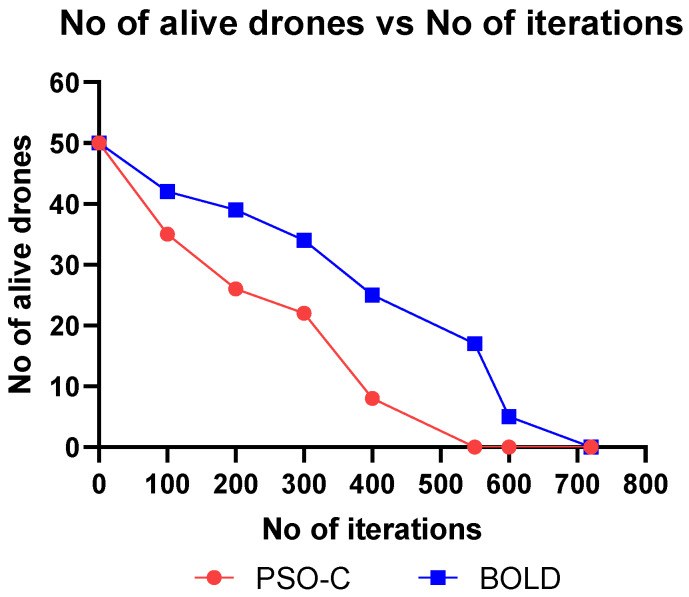
Number of alive drones vs iterations.

**Figure 15:**
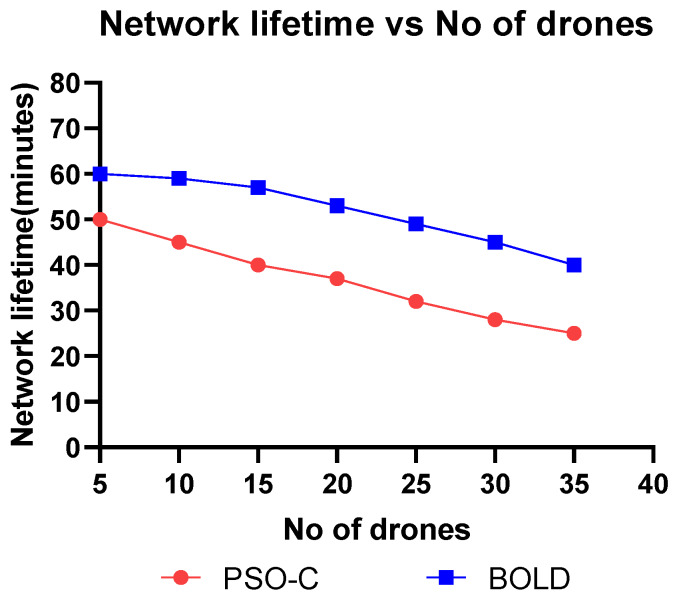
Network lifetime vs number of drones.

In ad-hoc networks, the network lifetime can be defined in several ways like, when 50% of the nodes are dead, or 100% of the nodes are dead. Drones are more energy-constrained, and even a single drone can perform operations alone. Hence, here we considered the iterations until the death of the last node. The simulation was performed for comparing the network lifetime in terms of iterations varying from 0 to 800 iterations, as shown in Figure 14 and in terms of the number of drones ranging from five to 35 as shown in Figure 15. The tabular values are shown in Table 6 and Table 7, respectively. From Figure 14 and Figure 15, it is observed that the network lifetime of BOLD is more than PSO-C, because in BOLD apart from residual energy, the drone which is closer to the base station and all other drones in the network is selected as a CH, whereas in PSO-C the leader election is based only on the residual energy. From the graph shown in Figure 15, it is clear that the network lifetime of BOLD is 15% higher than the PSO-C.

**Figure 16:**
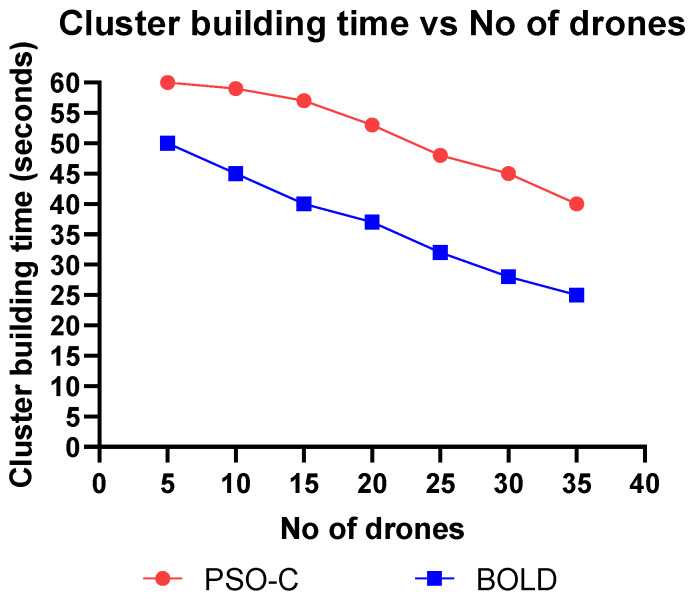
Cluster building time vs number of drones.

By using the fitness value of each node, the cluster is built, and the time is taken to form a cluster is called cluster building time. The cluster building time is the measure of the algorithmic complexity of the proposed algorithm. A high cluster building time denotes that the algorithm consumes more energy, in turn affecting the network lifetime. Figure 16 shows that the cluster building time of BOLD is 5.2% less than the PSO-C. The tabular values are shown in Table 8.

From the implementation results, it is clear that the energy consumption of the BOLD algorithm is 0.5 % lesser than the PSO-C, which gives a higher lifetime of 15% for the drones using BOLD when compared to those using PSO-C.

## 5. Conclusions

In this paper, an efficient approach has been proposed for leader election among multiple drones using bio-inspired optimization techniques. The stimulation has been performed considering the three-dimensional position (latitude, longitude, and altitude) of the drones rather than the two-dimensional structure, as proposed in earlier research. An enhanced algorithm for the election of the efficient cluster head and for the formation of clusters called the BOLD algorithm has been presented. The BOLD algorithm elects the CH among multi drones based on specific parameters such as distance and residual energy at different time periods. As per the mission requirement, the drones divide themselves into clusters based on their residual energy. The cluster is divided in such a manner that average residual energy for each cluster is equal. The leader election also takes place in these clusters.

The PSO-C algorithm, where the fitness value is based only on the residual energy has been compared with the BOLD algorithm, where the fitness value has been calculated based on both the residual energy and the distance between the drones, and the results that we have found from this study depict that the proposed BOLD algorithm is more efficient than the former in terms of extending the network lifetime by reducing communication energy consumption.

In the future, the transmission range and transmission frequency should be considered when the drones are communicating with each other. It will play a vital role in the election of the leader since, if the transmission range is very less or very high, it may lead to inefficient network communication. Moreover, the algorithm can be tested in the real-world environment with the help of drone simulators like X-PLANE.

## Figures and Tables

**Table 1:** Network parameters and specifications for BOLD.

Parameters	Symbol	Value
Latitude	λ	10.000° N–25.500° N
Longitude	φ	80.000° E–95.500° E
Altitude	alt	0.3–0.7 km
Velocity of drone	v	50–70 mph
Target area	a	500 × 500 m^2^
Residual energy	$R_{energy}$	11.1 V Transmitter Pack 27.75 Wh
Energy dissipated	$E_{elec}$	0.0000005 nJ/bit
Amplifier energy	$E_{amp}$	0.00000000001 pJ/bit/sq.m
Transmission Range	$t_{range}$	1 mile
Packet length	l	4000 bits
Number of drones	n	5, 10, 15, 20, 25, 30, 35
Number of clusters	$N_{c}$	2
Number of iterations	$N_{i}$	0–800

**Table 2:** Drone Parameters considered for implementation.

Drone	1	2	3	4	5
**at**	7.9426	12.9427	14.9428	11.9429	9.9425
**Long**	81.1366	80.1366	81.1367	81.1365	80.1364
**Alt(km)**	0.301	0.4983	0.3999	0.4121	0.35
**R_energy_ (J)**	2095	2146	2493	2575	2032

**Table 3:** The calculated values of distance and $B_{opt}$ attained after the first iteration.

Drone	1	2	3	4	5
**Dist (km)**	9582.5939	9307.8798	11,018.12557	12,359.87325	14,377.20102
$B_{opt}$	2964.02	2934.58	3450.13	3759.47	3891.44

**Table 4:** Residual energy (J) vs. number of iterations.

Number of Iterations	Residual Energy (J)	
	PSO-C	BOLD
0	9.9	9.9
50	8.4	8.7
100	7.1	8
150	6.3	7.4
200	5.1	6.5
250	3.9	5.1
300	2.4	4.3

**Table 5:** Energy consumption (J) vs the number of drones (1 unit = 10^4^ Joules).

No. of Drones	Energy Consumption (×10^4^J)	
	PSO-C	BOLD
5	1.7	1.3
10	2.3	1.9
15	2.8	2.4
20	3.3	2.9
25	4.1	3.5
30	4.9	4.1
35	5.6	4.9

**Table 6:** Number of alive drones vs number of iterations.

Number of Iterations	Number of Alive Drones	
	PSO-C	BOLD
0	50	50
100	35	42
200	26	39
300	22	34
400	8	25
550	0	17
600	0	5
720	0	0

**Table 7:** Network lifetime vs number of drones.

Number of Drones	Network Lifetime (Min)	
	PSO-C	BOLD
5	50	60
10	45	59
15	40	57
20	37	53
25	32	49
30	28	45
35	25	40

**Table 8:** Cluster building time vs the number of drones.

Number of Drones	Cluster Building Time (s)	
	PSO-C	BOLD
5	60	50
10	59	45
15	57	40
20	53	37
25	49	32
30	45	28
35	40	25
